# Clarifying the degradation process of luminescent inorganic perovskite nanocrystals†

**DOI:** 10.1039/d4ra07548j

**Published:** 2024-12-10

**Authors:** Yong Bin Kim, Sung Hun Kim, Yong-Ryun Jo, Sang-Youp Yim, Sang-Hyun Chin, Kyoung-Duck Park, Hong Seok Lee

**Affiliations:** a Department of Physics, Research Institute Physics and Chemistry, Jeonbuk National University Jeonju 54896 Republic of Korea hslee1@jbnu.ac.kr; b Department of Physics, Pohang University of Science and Technology (POSTECH) Pohang 37673 Republic of Korea parklab@postech.ac.kr; c Advanced Photonics Research Institute, Gwangju Institute of Science and Technology Gwangju 61005 Republic of Korea; d Department of Physics and van der Waals Materials Research Centre, Yonsei University Seoul 03722 Republic of Korea sanghyunchin@yonsei.ac.kr

## Abstract

Metal halide perovskites have emerged as highly promising materials for a range of optoelectronic applications. However, their sensitivity to environmental factors, particularly air moisture, presents significant challenges for both reliable research and commercialization. Moisture-induced degradation is a major issue due to the ionic nature of perovskites, which significantly impacts their luminescent properties. Despite extensive research efforts focusing on device applications, a comprehensive understanding of the degradation mechanisms in perovskites remains limited, largely due to their intrinsic ionic characteristics. In this work, we perform an in-depth analysis of the degradation process in perovskite nanocrystals (NCs) synthesized with varying reaction times, exploring the correlation between their optical and structural properties. Our findings reveal that perovskite NCs with larger crystal sizes exhibit greater stability in ambient air, attributed to their lower surface-to-volume ratio. These insights offer a deeper understanding of the relationship between perovskite NC degradation and their optical performance, contributing to advancements in the field of perovskite-based light-emitting technologies.

## Abstract 2

The size-dependent degradation process of inorganic CsPbBr_3_ perovskite nanocrystals in ambient air is revealed with structural and optical analysis upon long term air exposure.

## Graphical abstract

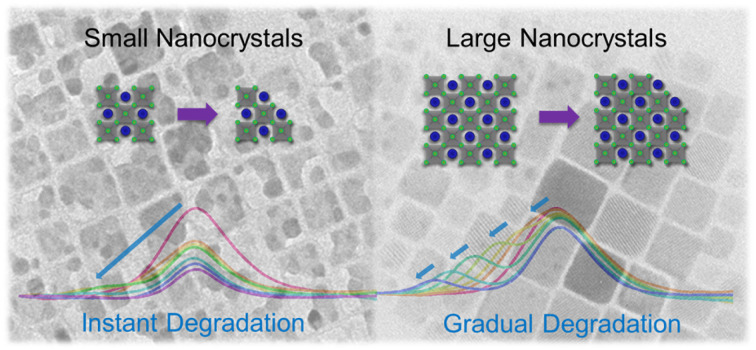

## 1. Introduction

Metal halide perovskites (MHPs) have garnered significant attention for their exceptional and tunable optoelectronic properties, positioning them as highly promising material platforms for applications, such as solar cells, light-emitting diodes (LEDs), and lasers.^1–11^ Extensive research efforts have focused on enhancing the optoelectronic performance of MHPs by leveraging their intrinsic ionic nature. Notably, the band gap of MHPs can be easily tuned through the selection of tailored ionic compositions, a capability that has been explored since the early stages of MHP research.^10,12,13^ For instance, Jeon *et al.* introduced formamidinium cation and bromide anion (Br^−^) into the prototypical methylammonium lead iodide (CH_3_NH_3_PbI_3_) perovskite structure, achieving record-breaking photon-to-electron power conversion efficiency and improved stability in solar cells.^14^ Currently defect-passivated MHPs are regarded as some of the most cost-effective and efficient photovoltaic materials, with applications in both standalone commercial devices and as top cells in tandem photovoltaics.^5,15^

In light-emitting applications, room-temperature operating LEDs and optically pumped lasers were first reported in 2014 by research groups from Cambridge and Valencia.^16–18^ The following year, Kim *et al.* demonstrated organic–inorganic hybrid perovskite LEDs featuring a sharp green emission (full width at the half maximum, FWHM ≈ 20 nm) using CH_3_NH_3_PbBr_3_ as the light-emissive layer. This work achieved high luminance and device efficiency, marking a significant milestone in the development of perovskite LEDs. They further realized multi-coloured LEDs by substituting Br^−^ ions with I^−^ ions and Cl^−^ ions (CH_3_NH_3_PbCl_*x*_Br_*y*_I_3−*x*−*y*_), successfully covering the entire visible spectrum.^19^ In a subsequent achievement by the same research group, highly efficient, bright, and stable perovskite LEDs were demonstrated, achieving a maximum brightness of ∼470 000 cd m^−2^, a maximum external quantum efficiency of 28.9%, and a half-lifetime of 520 h at 1000 cd m^−2^ (with an estimated half-lifetime >30 000 h at 100 cd m^−2^).^20^ These performance metrics meet commercial standards for display technology, and even greater efficiencies can be achieved when integrating these perovskite LEDs into tandem structures with traditional organic LEDs.^6^

It is possible that this superiority in efficiency may be attributed to mobile ions within MHP films, which assist in charge extraction or injection in optoelectronic devices.^2,21,22^ When an electrical bias is applied, these mobile ions redistribute to minimize the electric field within the perovskite layers, accumulating at the interfaces with neighbouring layers. This ion accumulation enhances the electric field near the interface and reduces charge-injection barriers, thus increasing the injection current in LEDs. However, this also leads to instability in humid environments due to the strong interaction between highly polar H_2_O molecules and the ions that within MHPs.^23^ The strength of the H_2_O-ion interaction results in degradation or re-crystallization of MHPs. While defect-induced ion migration can be mitigated through defect passivation, a thorough understanding of the degradation process in MHPs is essential for developing effective passivation strategies.^2,7^ In light of this, here we perform a detailed analysis of the degradation process of perovskite nanocrystals (NCs) under ambient conditions. For this study, CsPbBr_3_ NCs are employed due to their photoluminescent (PL) properties, which allow for the detection of intensity changes and size-dependent shifts in the PL peak. This enables the evaluation of degradation by correlating the PL characteristics of CsPbBr_3_ NCs and correlated with visual observations from transmission electron microscopy (TEM) results.

## 2. Experimental details

### 2.1 Synthesis

CsPbBr_3_ NCs with oleyl amine and oleic acid (OA) as capping ligands were fabricated by using hot injection method. To prepare the Cs-oleate solution, 391 mg of Cs_2_CO_3_, 1.27 mL of OA, and 18.73 mL of 1-octadecene (ODE) were added to a 100 mL three-necked flask. The mixture was placed under vacuum at 120 °C for 1 h and then heated at 160 °C under N_2_ flow.

In a separate three-necked flask, 149 mg of PbBr_2_ and 24 mL of ODE were placed under vacuum at 120 °C for 1 h and then 1 mL of OA and 3 mL of oleyl amine were injected into the three-necked flask at this temperature. Once the PbBr_2_ salt was fully dissolved, the temperature was raised to 180 °C, and 2 mL of preheated Cs-oleate stock solution was rapidly injected into the PbBr_2_ solution. Subsequently, the samples were obtained varying the reaction time. The crude solution was purified using *tert*-butanol and dispersed in toluene for further characterization. Prior to the subsequent measurements, CsPbBr_3_ NC samples are aged at room temperature and 45% relative humidity for varying durations.

### 2.2 Characterization

PL spectra were obtained by using inverted optical microscope (Nikon Eclipse Ti) with a 100× objective lens (NA ×0.9, Nikon). A hyperspectral imager (Photon *etc.*, IM000240001) was placed in front of the EMCCD (iXon3, DU-897E-C00-# BV), and the sample was excited using a 405 nm laser. Time-resolved photoluminescence (TRPL) measurements were conducted using the second harmonic generation at 400 nm from a mode-locked Ti:Sapphire laser (Chameleon Ultra II, Coherent). To study exciton dynamics, the excitation pulse frequency was reduced to a repetition rate of 1 MHz using a pulse picker (9200 series, Coherent Inc.), with a pulse duration of 200 fs. The emitted fluorescence was focused into the entrance slit of a 300 mm spectrograph (Acton SpectraPro 2300i, Princeton Instruments), which provides a spectral resolution of approximately 1 nm. Photoluminescence decay was then precisely monitored using a picosecond streak camera (C11200, Hamamatsu Photonics).

The crystalline structure and morphology of the CsPbBr_3_ NCs were investigated by using a FETEM (Tecnai G2, F30 S-Twin, 300 keV, Thermo Fisher Scientific, Waltham, MA, USA).

## 3 Results and discussions

**Fig. 1:**
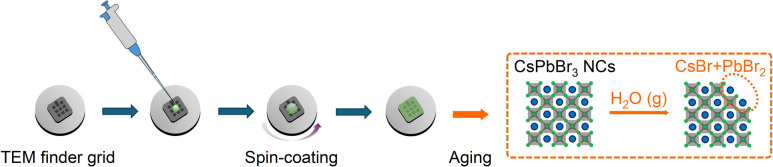
Schematic illustration of fabrication process and expected influence from humidity exposure.

To investigate the effect of moisture-induced degradation, CsPbBr_3_ NCs are synthesized with different reaction times: 5 seconds (P1), 10 minutes (P2), and 60 minutes (P3). After the synthetic process, the products are spin-coated on grids for further study using TEM as depicted in Fig. 1. It is noteworthy that the finder grids enable visual confirmation of the degradation of CsPbBr_3_ NC samples over time by ensuring ident spot of imaging. The PL properties of each pristine CsPbBr_3_ NCs are shown in Fig. S1 (ESI).† The PL peaks of the freshly prepared P1, P2, and P3 are located at 519, 522, and 527 nm respectively, confirming a clear size dependent quantum confinement effect. This will be discussed further with the obtained TEM images, which demonstrate a clear difference in the NCs size.

**Fig. 2:**
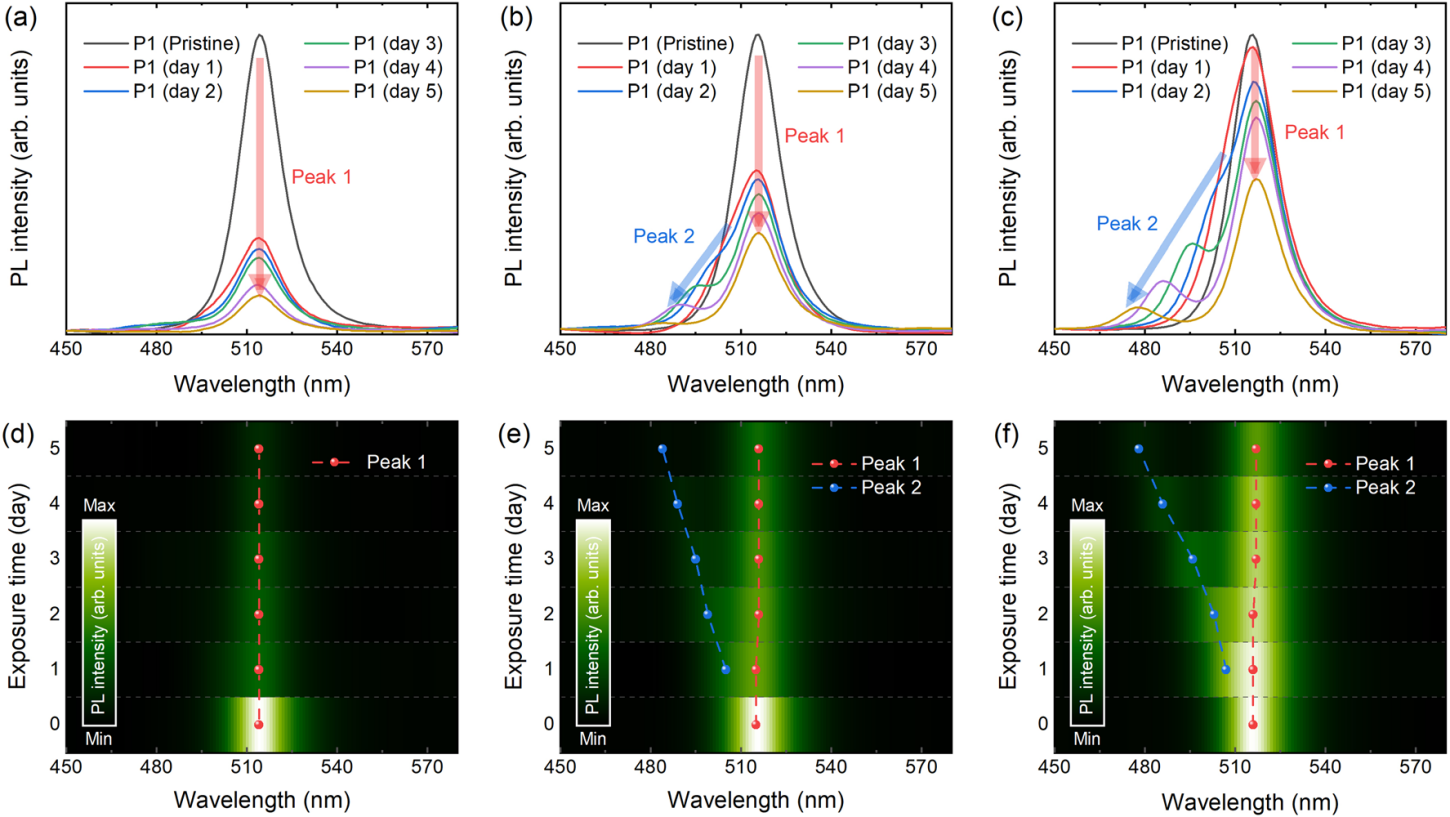
Steady state photoluminescence (PL) properties of the CsPbBr_3_ nanocrystals (NCs). PL spectra upon aging time of (a) P1, (b) P2, and (c) P3. (d–f) Contour plot of PL spectra with respect to the aging time with highlighted PL peaks.

With these CsPbBr_3_ NCs, firstly the PL property upon degradation is investigated. By collecting the signal of luminescence over 5 days, a clear trend in the PL spectra is observed, as shown in Fig. 2a–c. The intensity of the initial PL peak (Peak 1) of P1 is decreased below half of the initial intensity within 1 day, however the intensity of Peak 1 remained better with the samples synthesized with prolonged reaction time (Fig. S2, ESI†). Interestingly, another PL peak (Peak 2) in the lower wavelength-regime is detected in both P2 and P3 (Peak 2 of P1 is magnified in Fig. S3, ESI†). Peak 2 of P1 is detected at approximately 480 nm after 2 days, exhibiting a relatively weak intensity. Meanwhile, the secondary PL peaks from P2 and P3 undergo a gradual blue shift, resulting in a broadening of the emission spectrum, as illustrated in Fig. 2d–f. Since perovskite light-emitters are emerging as promising candidates for meeting Rec. 2020 standards in ultra-high-definition display technology, maintaining sharp emission spectra is highly desirable.^2,13,24^ Thus, it is possible to comment that clarifying the origin of this spectrum-broadening is a crucial step for preventing it and finally realizing perovskite display with high colour gamut.

**Fig. 3:**
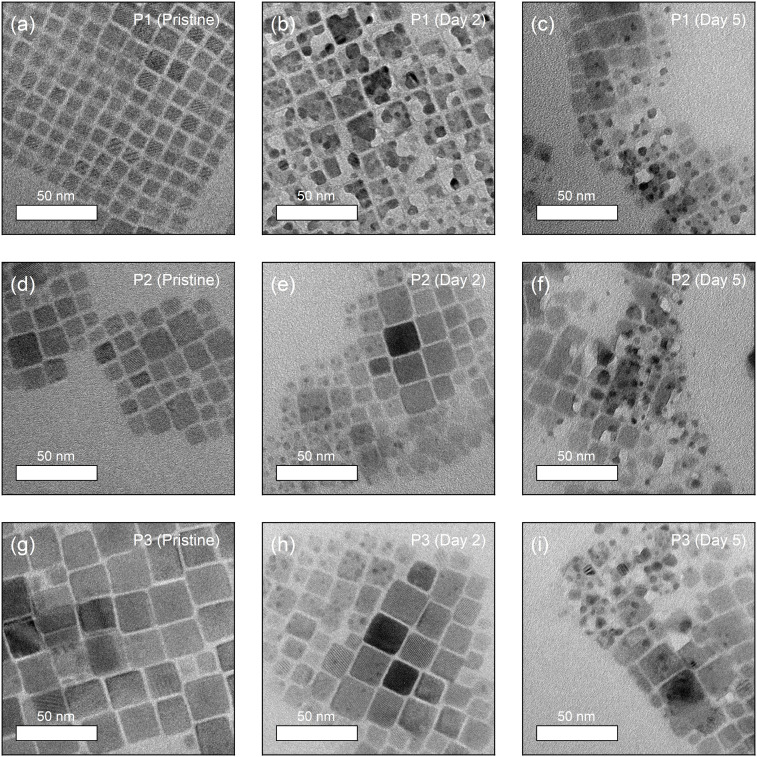
TEM images of (a) fresh reference P1, (b) P1 exposed for 2 days, and (c) P1 exposed for 5 days. TEM images of (d) fresh reference P2, (e) P2 exposed for 2 days, and (f) P2 exposed for 5 days. TEM images of (g) fresh reference P3, (h) P3 exposed for 2 days, and (i) P3 exposed for 5 days.

Recent literature suggests two plausible scenarios to explain the independent, blue-shifted photoluminescence peak (Peak 2): (i) decomposed perovskites form precursors that self-confine, leading to a quantum confinement effect, or (ii) the presence of H_2_O promotes the formation of the blue-emissive two-dimensional byproduct, CsPb_2_Br_5_.^25^ Since both scenarios explain the blue emission observed after degradation, additional insight into this phenomenon is required. To investigate further, we performed TEM imaging, tracked over 5 days. As shown in the images, degradation is visually confirmed according to the following timeline for each sample: Fig. 3a pristine P1 – 3b after 2 days – 3c after 5 days, 3d pristine P2 – 3e after 2 days – 3f after 5 days, and 3g pristine P3 – 3h after 2 days – 3i after 5 days. Comparing the fresh reference samples, the initial size of perovskite NCs are in a good agreement with the trend of PL spectra as mentioned, assuming quantum confinement effect. As the air-exposure time increases, several black dots appear, particularly in P1, which has relatively smaller crystal size, showing rapid formation of these black dots within the first 2 days. This might be explained by the high surface-to-volume ratio that reinforces exposure to humid air. However, there is no clear difference in the image of P1 after 5 days, despite the rapid decrease in PL intensity (as shown above), which saturates after 4 days. The correlation between the TEM images and PL measurement results suggests that the decomposition to CsBr + PbBr_2_ may suppress further degradation of CsPbBr_3_ NCs. In 2014, Chen *et al.* reported a comparable trend for CH_3_NH_3_PbI_3_, specifically self-induced passivation.^26^ They proposed a method for effectively passivating CH_3_NH_3_PbI_3_ films. It has been shown that the presence of PbI_2_ species in the grain boundaries upon thermal degradation led to a successful passivation that controls the carrier behaviour along the heterojunctions, since PbI_2_ species has wider band gap.

**Fig. 4:**
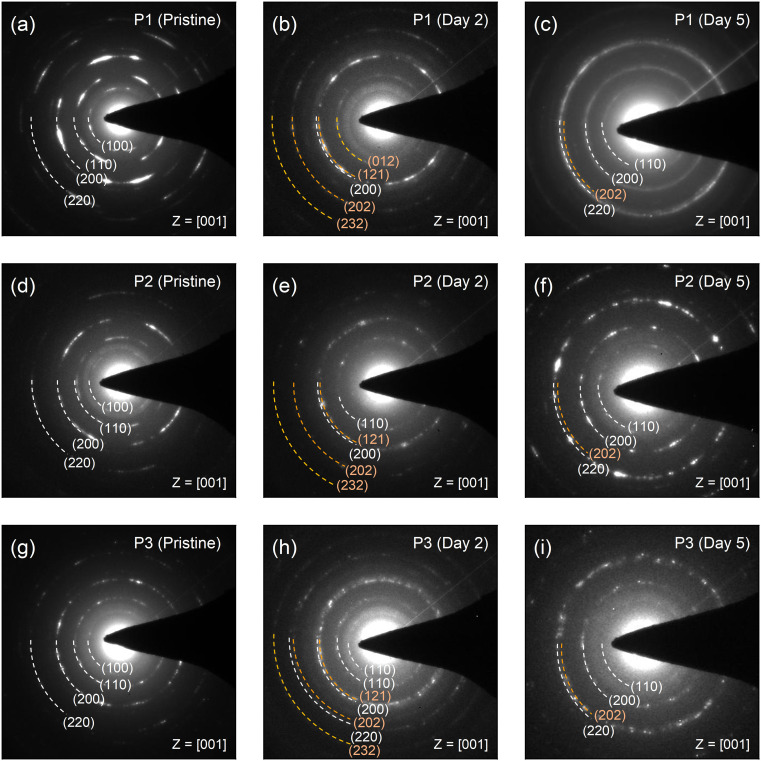
Selected area electron diffraction (SAED) patterns of (a) fresh reference P1, (b) P1 exposed for 2 days, and (c) P1 exposed for 5 days. SAED patterns of (d) fresh reference P2, (e) P2 exposed for 2 days, and (f) P2 exposed for 5 days. SAED patterns of (g) fresh reference P3, (h) P3 exposed for 2 days, and (i) P3 exposed for 5 days.

Similarly, decomposition induced formation of PbBr_2_ might protect CsPbBr_3_ NCs against moisture in air. To confirm this phenomenon and verify the presence of PbBr_2_, a series of studies using selected area electron diffraction (SAED) patterns is performed as shown in Fig. 4. P1–P3 exhibit (100), (110), (200), and (220) signals, purely from CsPbBr_3_ in the SAED results right after fabrication. However, with increasing exposure to humid air, the samples begin to show (012), (121), (202), and (232) signals from PbBr_2_.

By this clear result, it is verified that the blue emission of PL spectra originated from PbBr_2_, yet not related to 2D CsPb_2_Br_5_. In addition, supporting experimental and theoretical studies by Fausia *et al.* reveal that H_2_O molecules catalyse the decomposition of CsPbBr_3_ into PbBr_2_ and CsBr through a two-step mechanism involving “surface adsorption” and “subsequent intercalation”.^27^ Initially, water molecules adsorb on the (100) surface of CsPbBr_3_ where the hydroxyl groups interact with the exposed Cs and Pb atoms. This adsorption weakens the structural integrity of the perovskite, facilitating the penetration of water molecules into the crystal lattice. Theoretical insights presented in this study reveal that intercalated water molecules form strong interactions with Cs and Pb atoms, leading to lattice compression and a significant volume contraction of approximately 6.57%. This process disrupts the Cs–Br ionic interactions, which play a vital role in maintaining the structural stability of the perovskite. The redistribution of charges caused by water intercalation eventually leads to the dissociation of CsPbBr_3_ into its degradation-products. Pearson's hard-soft acid–base theory further explains this mechanism, as Cs^+^ and Pb^2+^ cations preferentially form stable salts with bromide ions, which accelerates the decomposition in a moisture-rich environment. This anticipation is in the same vein as the result of PL spectra and TEM images. It is highly plausible that the intercalated H_2_O molecules degrade CsPbBr_3_ NCs from inside and separate into smaller NCs, resulting in additional blue shifted PL peaks and dots in electron microscopic images. Notably, the results with P1 after 5 days in Fig. 4c do not demonstrate PbBr_2_-related signals but only from CsPbBr_3_, which might stem from the CsPbBr_3_ produced by moisture-assisted reaction of decomposed precursors.^23^ This demonstrates a crucial difference between selected inorganic CsPbBr_3_ perovskite material and organic–inorganic metal halide perovskites. For instance, methylammonium-based MHPs (CH_3_NH_3_PbX_3,_ X = halide anions) permanently lose gas phase products during decomposition, such as “CH_3_NH_2_ + HX pair” or “CH_3_X + NH_3_ pair”.^28^ Thus, methylammonium-based MHPs might require excess methylammonium halide components, which causes difficulty in observation of blue-shifted PL from smaller perovskites in stoichiometrically synthesized system such as perovskite NCs.

**Fig. 5:**
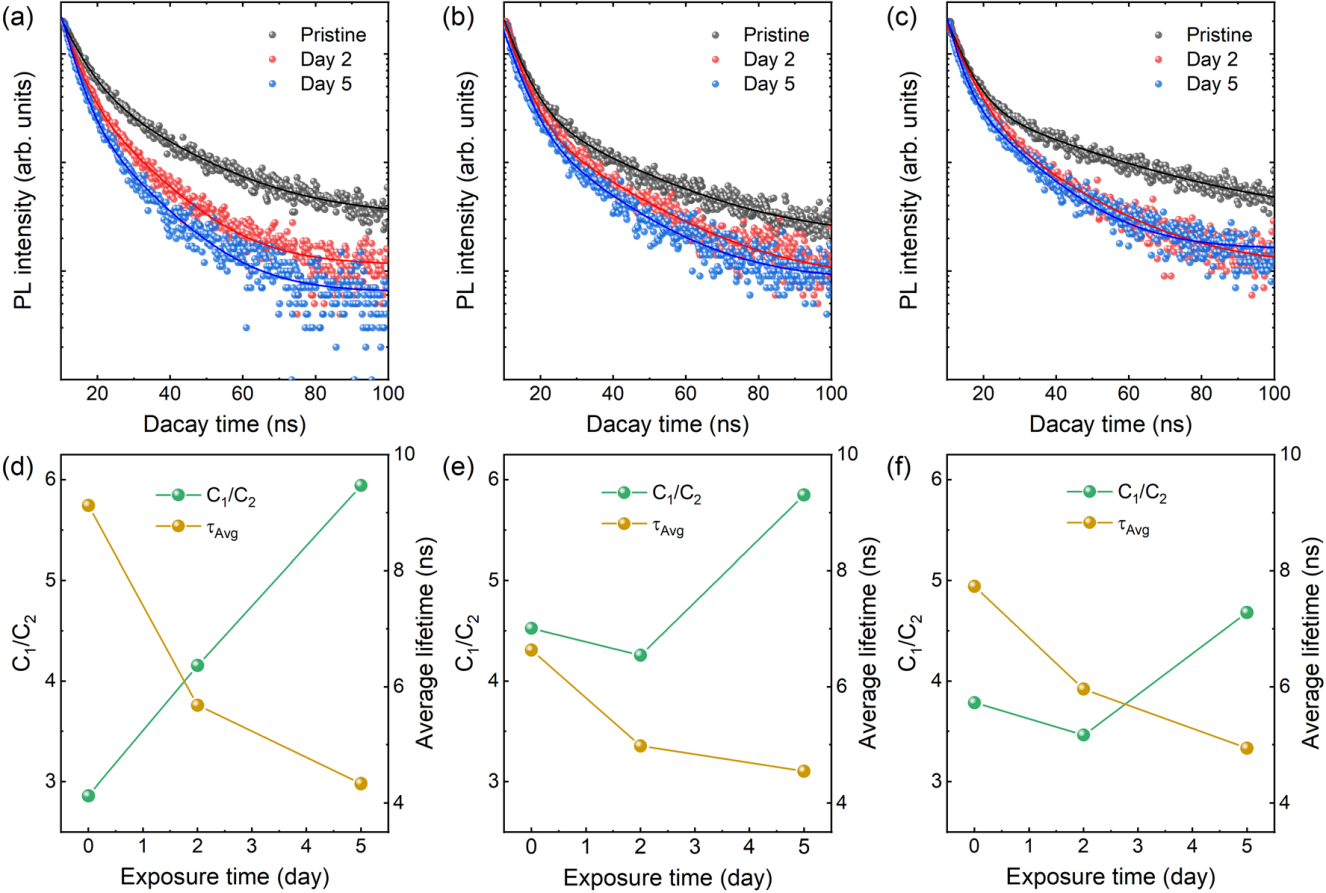
Time-resolved photoluminescence (TRPL) spectra of (a) P1, (b) P2, and (c) P3. Solid lines indicate fitting curves with a biexponential function. Relative weight fractions (*C*_1_/*C*_2_) and average exciton lifetime of (d) P1, (e) P2, and (f) P3.

For a further investigation elucidating the impact of degradation and PbBr_2_-formation on recombination dynamics, TRPL spectra are obtained for the air-exposed CsPbBr_3_ NCs. By collecting TRPL signals for 5 days, a consistent trend is confirmed. As air-exposure time increase, the lifetime decreases for P1, P2, and P3, as shown in Fig. 5a–c. The shortening of PL lifetime is usually attributed to an increase in non-radiative recombination pathway.^29^ The TRPL results are deconvoluted using a bi-exponential decay model, described as follows:*I* = *C*_1_ exp(−*t*/*τ*_1_) + *C*_2_ exp(−*t*/*τ*_2_)*τ*_Avg_ = (*C*_1_*τ*_1_^2^ + *C*_2_*τ*_2_^2^)/(*C*_1_*τ*_1_ + *C*_2_*τ*_2_)Here, “*I*” represents the intensity of emission, and *C*_1_ and *C*_2_ are the amplitudes associated with the decay components. The shorter (fast) lifetime (*τ*_1_) corresponds to excitonic recombination, which includes contributions from defect or trap states, while the longer (slow) lifetime *τ*_2_ corresponds to radiative recombination and *t* denotes the time variable.^30^ The PL lifetimes and fractional intensities of CsPbBr_3_ NCs before and after air exposure are summarized at Table S1 (ESI†). Fig. 5d–e show the average exciton lifetime (*τ*_Avg_) and the relative weight fractions (RWFs, *C*_1_/*C*_2_) over the air-exposure time. It is clearly shown that average PL lifetime decreases, and the RWFs increase with prolonged air-exposure. The increase in RWFs suggests a rise in the number of non-radiative pathways, leading to the quenching of PL intensity. Recent literature attributes this increase in non-radiative recombination pathways to the formation of surface defects, typically associated with the decomposition of CsPbBr_3_ NCs into CsBr and PbBr_2_.^30,31^ The decomposition observed in our TEM measurements strongly supports this interpretation.

## 4 Conclusions

In summary, the degradation of perovskite NCs was visually confirmed using TEM and closely correlated with the PL properties of inorganic perovskite NCs synthesized with varying reaction times. Through a comprehensive series of measurements, it was established that the low surface-to-volume ratio of the NCs, along with the presence of surrounding PbBr_2_, plays a key role in preventing perovskite decomposition. This work is especially significant as it highlights the importance of carefully designing additive engineering and other techniques aimed at improving perovskite quality. Such approaches must consider critical factors, including the formation energy of the final product and the specific types of defects present.

## Data availability

The data that support the findings of this study are available from the corresponding authors upon reasonable request.

## Conflicts of interest

There are no conflicts to declare.

## Supplementary Material

RA-014-D4RA07548J-s001

## References

[cit1] Xu Z., Chin S.-H., Park B.-I., Meng Y., Kim S., Han S., Li Y., Kim D.-H., Kim B.-S., Lee J.-W., Bae S.-H. (2024). Next Materials.

[cit2] Fakharuddin A., Gangishetty M. K., Abdi-Jalebi M., Chin S.-H., bin Mohd Yusoff A. R., Congreve D. N., Tress W., Deschler F., Vasilopoulou M., Bolink H. J. (2022). Nat. Electron..

[cit3] Chin S. H., Cortecchia D., Forzatti M., Wu C. S., Alvarado-Leaños A. L., Folpini G., Treglia A., Kalluvila Justin I. A., Paliwal A., Cho C., Roldán-Carmona C., Sessolo M., Petrozza A., Bolink H. J. (2024). Adv. Opt. Mater..

[cit4] Grätzel M. (2017). Acc. Chem. Res..

[cit5] Park J., Kim J., Yun H. S., Paik M. J., Noh E., Mun H. J., Kim M. G., Shin T. J., Il Seok S. (2023). Nature.

[cit6] Lee H. D., Woo S. J., Kim S., Kim J., Zhou H., Han S. J., Jang K. Y., Kim D. H., Park J., Yoo S., Lee T. W. (2024). Nat. Nanotechnol..

[cit7] Kim Y. H., Kim S., Kakekhani A., Park J., Park J., Lee Y. H., Xu H., Nagane S., Wexler R. B., Kim D. H., Jo S. H., Martínez-Sarti L., Tan P., Sadhanala A., Park G. S., Kim Y. W., Hu B., Bolink H. J., Yoo S., Friend R. H., Rappe A. M., Lee T. W. (2021). Nat. Photonics.

[cit8] Kim Y. H., Park J., Kim S., Kim J. S., Xu H., Jeong S. H., Hu B., Lee T. W. (2022). Nat. Nanotechnol..

[cit9] Chin S. H. (2024). Discover Appl. Sci..

[cit10] Sutherland B. R., Sargent E. H. (2016). Nat. Photonics.

[cit11] Forzatti M., Chin S. H., Hernández-Fenollosa M. A., Sessolo M., Tordera D., Bolink H. J. (2024). Adv. Opt. Mater..

[cit12] Rudd P. N., Huang J. (2019). Trends Chem.

[cit13] Young-Hoon K., Himchan C., Tae-Woo L. (2016). Proc. Natl. Acad. Sci. U. S. A..

[cit14] Jeon N. J., Noh J. H., Yang W. S., Kim Y. C., Ryu S., Seo J., Il Seok S. (2015). Nature.

[cit15] Aydin E., Allen T. G., De Bastiani M., Razzaq A., Xu L., Ugur E., Liu J., De Wolf S. (2024). American Association for the Advancement of Science.

[cit16] Tan Z.-K., Moghaddam R. S., Lai M. L., Docampo P., Higler R., Deschler F., Price M., Sadhanala A., Pazos L. M., Credgington D., Hanusch F., Bein T., Snaith H. J., Friend R. H. (2014). Nat. Nanotechnol..

[cit17] Schmidt L. C., Pertegás A., González-Carrero S., Malinkiewicz O., Agouram S., Mínguez Espallargas G., Bolink H. J., Galian R. E., Pérez-Prieto J. (2014). J. Am. Chem. Soc..

[cit18] Deschler F., Price M., Pathak S., Klintberg L. E., Jarausch D.-D., Higler R., Hüttner S., Leijtens T., Stranks S. D., Snaith H. J., Atatüre M., Phillips R. T., Friend R. H. (2014). J. Phys. Chem. Lett..

[cit19] Kim Y. H., Cho H., Heo J. H., Kim T. S., Myoung N. S., Lee C. L., Im S. H., Lee T. W. (2015). Adv. Mater..

[cit20] Kim J. S., Heo J. M., Park G. S., Woo S. J., Cho C., Yun H. J., Kim D. H., Park J., Lee S. C., Park S. H., Yoon E., Greenham N. C., Lee T. W. (2022). Nature.

[cit21] Chin S. H., Mardegan L., Palazon F., Sessolo M., Bolink H. J. (2022). ACS Photonics.

[cit22] Mao P., Shan X., Li H., Davis M., Pei Q., Yu Z. (2022). ACS Appl. Electron. Mater..

[cit23] Chin S. H., Choi J. W., Woo H. C., Kim J. H., Lee H. S., Lee C. L. (2019). Nanoscale.

[cit24] Han T. H., Jang K. Y., Dong Y., Friend R. H., Sargent E. H., Lee T. W. (2022). Nature Research.

[cit25] Turedi B., Lee K. J., Dursun I., Alamer B., Wu Z., Alarousu E., Mohammed O. F., Cho N., Bakr O. M. (2018). J. Phys. Chem. C.

[cit26] Chen Q., Zhou H., Bin Song T., Luo S., Hong Z., Duan H. S., Dou L., Liu Y., Yang Y. (2014). Nano Lett..

[cit27] Fausia K. H., Nharangatt B., Vinayakan R. N., Ramesh A. R., Santhi V., Dhandapani K. R., Manoj T. P., Chatanathodi R., Jose D., Sandeep K. (2024). ACS Omega.

[cit28] Juarez-Perez E. J., Ono L. K., Uriarte I., Cocinero E. J., Qi Y. (2019). ACS Appl. Mater. Interfaces.

[cit29] Qiu J., Xue W., Wang W., Li Y. (2022). Dyes Pigm..

[cit30] Varnakavi N., Velpugonda J. L., Lee N., Nah S., Lin L. Y. (2024). Adv. Funct. Mater..

[cit31] Liu W., Yuan G., Zhang Y., Wang Q., Zhao S., Liu Z., Wei T., Wang J., Li J. (2019). J Mater Chem C Mater.

