# Supplementary material for: Clarifying the degradation process of luminescent inorganic perovskite nanocrystals
Source: RSC Adv. 2024 Dec 10;14(52):38378–84. doi: 10.1039/d4ra07548j (PMC11629105; doi:10.1039/d4ra07548j)
Supplement: RA-014-D4RA07548J-s001 [file RA-014-D4RA07548J-s001.pdf]

Supplementary Information (SI) for RSC Advances.
This journal is © The Royal Society of Chemistry 2024

Supplementary Information

Clarifying Degradation Process of Luminescent Inorganic Perovskite Nanocrystals

Yong Bin Kim [ab], Sung Hun Kim [a], Yong-Ryun Jo [c], Sang-Youp Yim [c], Sang-Hyun Chin*[d], Kyoung-Duck Park*[b] and Hong Seok Lee*[a]

a. Department of Physics, Research Institute Physics and Chemistry, Jeonbuk National University, Jeonju 54896, Republic of Korea. Email: hslee1@jbnu.ac.kr

b. Department of Physics, Pohang University of Science and Technology (POSTECH), Pohang 37673, Republic of Korea. Email: parklab@postech.ac.kr

c. Advanced Photonics Research Institute, Gwangju Institute of Science and Technology, Gwangju 61005, Republic of Korea

d. Department of Physics and van der Waals Materials Research Centre, Yonsei University, Seoul 03722, Republic of Korea. Email: sanghyunchin@yonsei.ac.kr

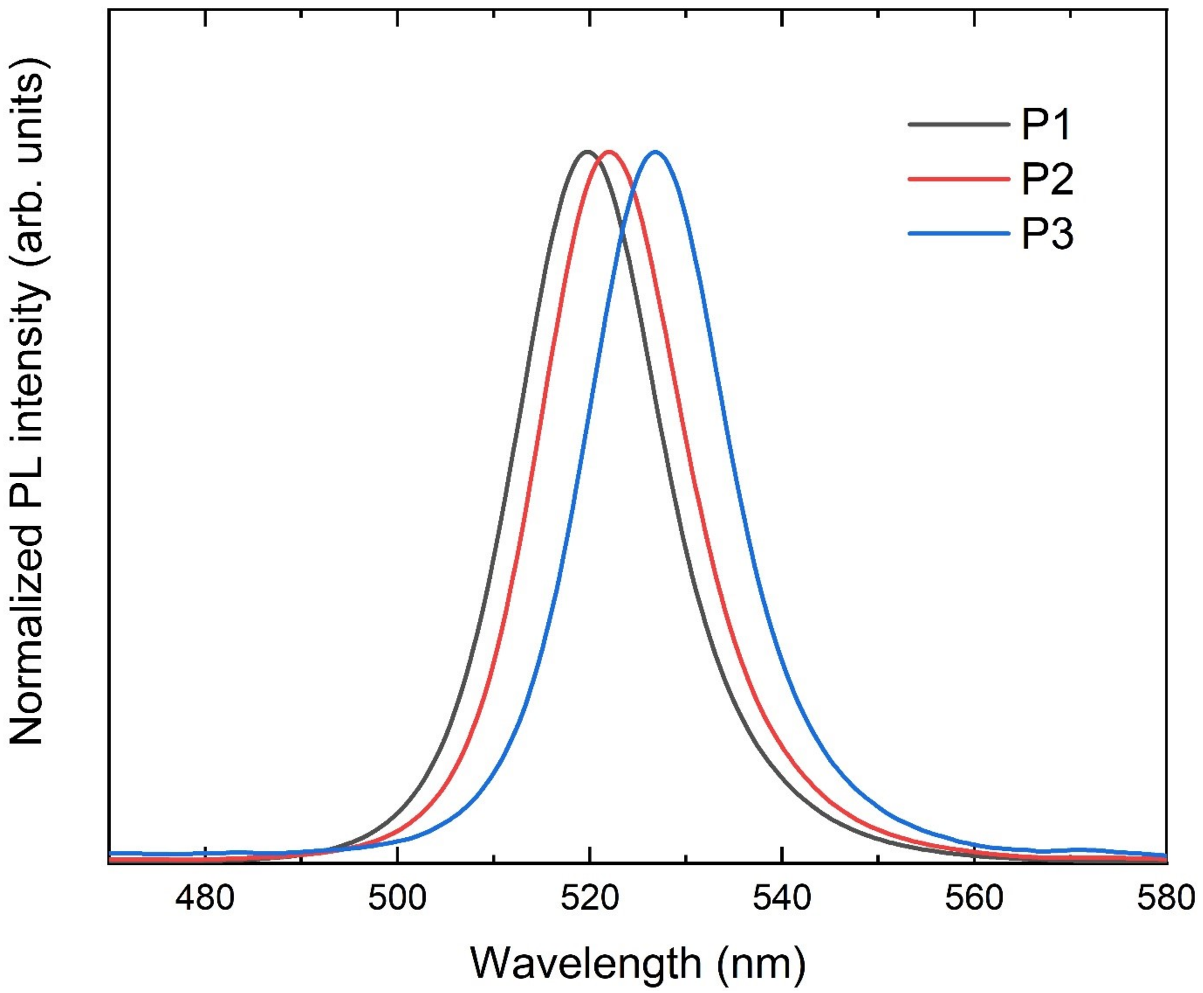

Fig. S1. Normalized PL spectra of pristine P1 (reaction time: 5 s), P2 (reaction time: 10 min), and P3 (reaction time: 60 min). The emission peaks are 519, 522, and 527 nm, respectively.

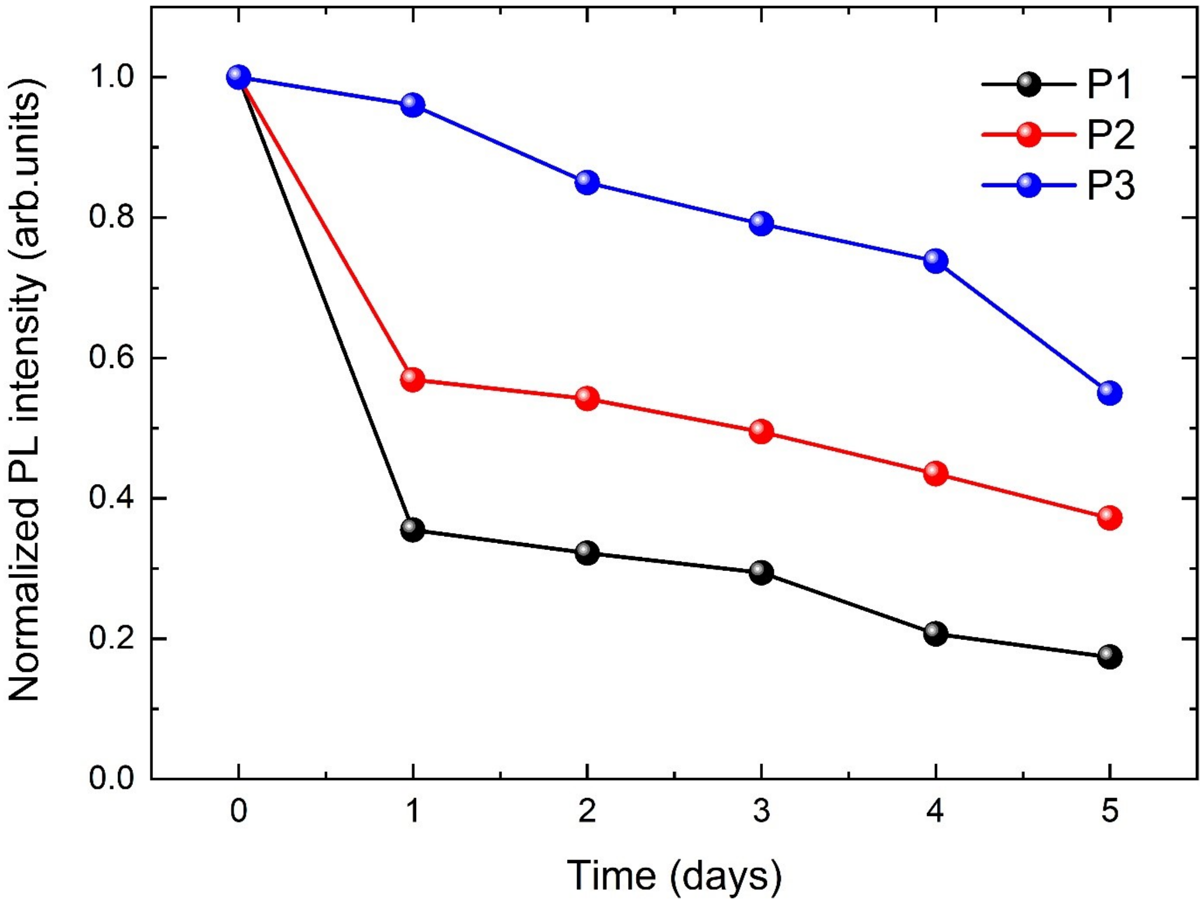

Fig. S2. Normalized PL intensity of P1, P2, and P3 on air exposure.

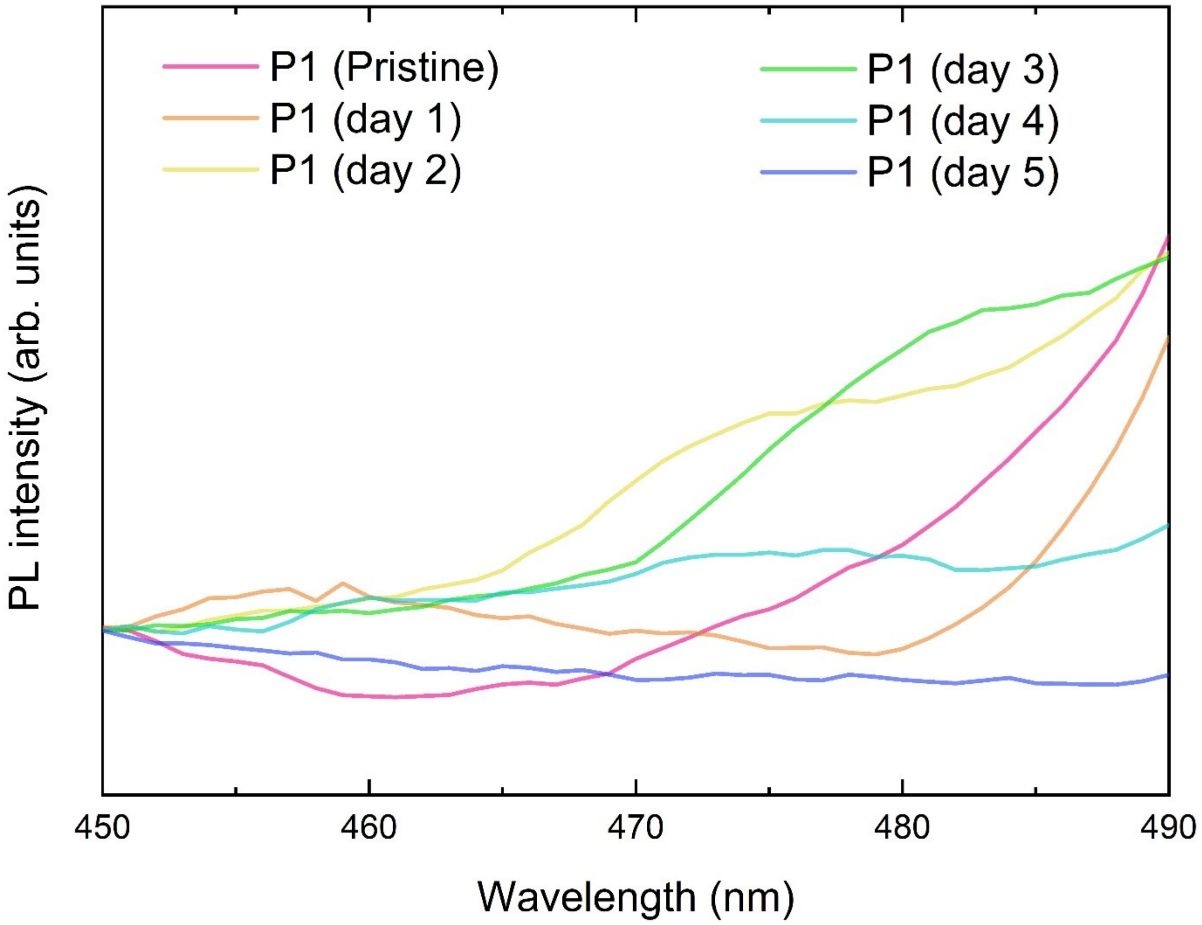

Fig. S3. Magnified PL spectra of P1 on air exposure.

Table S1 The average lifetime components and their relative amplitudes of P1, P2, and P3.

P1	τ_1 (ns)	τ_2 (ns)	C_1	C_2	τ_{Avg} (ns)
Pristine	4.736	21.659	0.741	0.259	9.123
Day2	3.622	14.271	0.806	0.194	5.683
Day5	3.058	11.946	0.856	0.144	4.334

P2	τ_1 (ns)	τ_2 (ns)	C_1	C_2	τ_{Avg} (ns)
Pristine	3.53	20.644	0.819	0.181	6.631
Day2	2.793	14.271	0.809	0.191	4.982
Day5	2.857	14.393	0.854	0.146	4.544

P3	τ_1 (ns)	τ_2 (ns)	C_1	C_2	τ_{Avg} (ns)
Pristine	3.211	24.852	0.791	0.209	7.733
Day2	3.348	15.006	0.776	0.224	5.96
Day5	2.793	15.006	0.824	0.176	4.945
